# Genes and pathways revealed by whole transcriptome analysis of milk derived bovine mammary epithelial cells after *Escherichia coli* challenge

**DOI:** 10.1186/s13567-024-01269-y

**Published:** 2024-02-01

**Authors:** Terhi Iso-Touru, Frank Panitz, Daniel Fischer, Minna K. Kyläniemi, Suvi Taponen, Jonna Tabell, Anneli Virta, Johanna Vilkki

**Affiliations:** 1 Natural Resources Institute Finland (Luke), Jokioinen, Finland; 2 Faculty of Veterinary Medicine, University of Helsinki, Helsinki, Finland; 3 Present Address: Turku Bioscience Centre, University of Turku and Åbo Akademi University, Turku, Finland

**Keywords:** Mastitis, infection, *Escherichia coli*, bovine, primary bovine mammary epithelial cells, transcriptome

## Abstract

Mastitis, inflammation of the mammary gland, is the costliest disease in dairy cattle and a major animal welfare concern. Mastitis is usually caused by bacteria, of which staphylococci, streptococci and *Escherichia coli* are most frequently isolated from bovine mastitis. Bacteria activate the mammary immune system in variable ways, thereby influencing the severity of the disease. *Escherichia coli* is a common cause of mastitis in cattle causing both subclinical and clinical mastitis. Understanding of the molecular mechanisms that activate and regulate the host response would be central to effective prevention of mastitis and breeding of cows more resistant to mastitis. We used primary bovine mammary epithelial cell cultures extracted noninvasively from bovine milk samples to monitor the cellular responses to *Escherichia coli* challenge. Differences in gene expression between control and challenged cells were studied by total RNA-sequencing at two time points post-challenge. In total, 150 and 440 (P_adj_ < 0.05) differentially expressed genes were identified at 3 h and 24 h post-challenge, respectively. The differentially expressed genes were mostly upregulated at 3 h (141/150) and 24 h (424/440) post-challenge. Our results are in line with known effects of *E. coli* infection, with a strong early inflammatory response mediated by pathogen receptor families. Among the most significantly enriched early KEGG pathways were the TNF signalling pathway, the cytokine-cytokine receptor interaction, and the NF-kappa B signalling pathway. At 24 h post-challenge, most significantly enriched were the Influenza A, the NOD-like receptor signalling, and the IL-17 signaling pathway.

**Supplementary Information:**

The online version contains supplementary material available at 10.1186/s13567-024-01269-y.

## Introduction

Mastitis, inflammation of the mammary gland, is the costliest disease in dairy cattle and the most important cause of antimicrobial use in dairy cattle. In Finland, the estimated average cost of clinical mastitis is 600€ per cow [1]. Mastitis is usually caused by bacteria, of which *Staphylococcus (S.) aureus*, non-aureus staphylococci (NAS), *Streptococcus dysgalactiae*, *Streptococcus uberis* and *Escherichia (E.) coli* are most frequently isolated from mastitic milk samples [2]. Mastitis can be expressed as subclinical without clinical signs, or clinical with signs varying from mild local signs to severe systemic signs [3]. The bacteria activate the mammary immune system in a variety of ways, and thereby influence the severity of the disease. Mastitis resistance in cattle is a genetically complex trait, with low to moderate heritabilities (ranging from 0.02 to 0.15 for clinical mastitis and milk somatic cell count) [4]. Attempts to improve mastitis resistance through breeding have not been very successful due to the low heritability for clinical mastitis and unfavourable genetic correlations with milk yield [4]. Understanding the molecular mechanisms that activate and regulate the mammary immune response would be central to the development of effective prevention of mastitis and methods for breeding mastitis resistance.

*Escherichia coli* is the leading cause of acute clinical mastitis in dairy cattle worldwide. A multi-country meta-analysis of microorganism species isolated from bovine milk samples revealed that the environmental pathogen *E. coli* was the most frequent pathogen species identified from milk of dairy cows having clinical mastitis [5]. The severity of mastitis caused by *E. coli* depends on several cow specific factors such as age of the cow, lactation stage and proximity of parturition (reviewed by [6]). Although non-antimicrobial approaches have been suggested to be the first option to treat mild to moderate *E. coli* mastitis, most cows with non-severe mastitis caused by *E. coli* are treated with antimicrobials within a week of onset of clinical mastitis [7].

The major cause for the strong inflammatory reaction causing the severe clinical symptoms by *E. coli* is considered to be the lipopolysaccharides (LPSs), a component of the cell wall of gram-negative bacteria [8]. The recognition of LPSs from *E. coli* is derived by mammary epithelial cells. Thus, during recent years, it has become clear that epithelial cells of the mammary gland not only synthesize and secrete milk but are crucial to initiate and cover the initial steps of the immune response [9]. In bovine primary Mammary Epithelial Cells (pbMECs), *E. coli* challenge has been shown to induce the expression of for example Toll-like receptor 2 (TLR2) and Toll-like receptor 4 (TLR4), and the cytokines Tumor Necrosis Factor-α, Interleukin-1α, Interleukin-6 and Interleukin-8, and activation of the NF-kappa B signalling pathway [10].

For several years various studies have aimed to understand the underlying molecular mechanisms in pathogenesis of mastitis to improve mastitis resistance in cattle. Brajnik and Ogorevc [11] have used a data integration approach to summarize findings from association, transcriptomic and mouse model studies and identify the most promising candidate loci for mastitis resistance. In all, the expression of 2300 candidate genes differed during mastitis compared to unaffected samples in eight transcriptomic studies reviewed by [11]. After combining all study types, they indicated 22 promising candidate genes. Still important questions remain how to exploit these findings in improving mastitis resistance.

Mammary epithelial cell responses have been studied using mammary epithelial cells derived from mammary biopsies (e.g. [12, 13]) but pbMECs derived from milk have rarely if at all been used for transcriptomic studies with pathogen challenge. Our primary aim was to investigate if milk derived epithelial cells have the potential to be used as a cell model to study mastitis resistance. We used pbMEC cultures extracted noninvasively from bovine milk samples to monitor the cells responses to pathogen challenge. By that, we wanted to identify the full set of genes and pathways affected by *E. coli* challenge at 3 and 24 h post-challenge using RNA transcriptome sequencing.

## Materials and methods

### Primary cell extraction

Milk from three healthy cows with no history of mastitis (Nordic Red Dairy cattle) was collected in mid-lactation by standard machine milking at the research barn at Natural Resources Institute Finland (Minkiö, FI) to sterile collection bottles and transported immediately to the laboratory. The protocol for pbMEC extraction from milk was adapted from Danowski et al. [14] and Hillreiner et al. [15] with some modifications. Approximately four litres of milk per animal were centrifuged 10 min at 1850 × *g*, RT without braking (Sorvall LYNX 6000 Superspeed Centrifuge/swinging bucket rotor BIOflex HC, Thermo Fisher Scientific).The pellets were re-suspended and washed three times (250 × *g*, 10 min, at RT without braking) in 20 mL of 37 °C washing medium (1 × HBSS (P04-50500, Pan Biotech, Germany), 1 M HEPES (L1612, Biochrom, UK)) containing 200 µg/mL of gentamicin sulfate (Pan Biotech, Germany), 10 µg/mL of amphotericin B (Biowest, France), 200 µg/mL of penicillin and 200 µg/mL of streptomycin (Biowest, France). After the last washing step, two filtration steps were performed using 100 µm and 40 µm pore size filters [cell strainer 100 µm, (VWR Collection) and cell strainer 40 µm (VWR Collection)]. The filtrate was centrifuged (250 × *g*, 10 min, at RT without braking) and suspended in a culture media consisting of DMEM F12-Ham (Biowest, France) supplemented with FBS (Gibco, Thermo Fisher Scientific, US), insulin-transferrin-selenite (ITS, Pan-Biotech, Germany), 100 µg/mL of gentamicin sulfate (Pan Biotech, Germany), 5 µg/mL of amphotericin B (Biowest, France), 100 µg/mL of penicillin and 100 µg/mL of streptomycin (Biowest, France).

The harvested pbMECs were cultured (5% CO_2_, 37 °C) approximately 14 days on 35 × 10 mm culture dish (Corning Inc, US, BioCoat™ Collagen I coated), detached with 0,25% trypsin/PBS solution and further cultured on 100 × 20 mm culture dish (Greiner Bio-One, Austria, Collagen I coated) until ~80% confluency (approximately 7 days) in the culture media described above. In the second passage at ~80% confluency, cells were harvested, counted with Countess II FL automated cell counter (Thermo Fisher Scientific, USA) and stored in freezing media (Bambanker DMSO Free, Nippon Genetics, Germany) in liquid nitrogen until pathogen challenge.

### Immunocytochemistry

The epithelial character of the harvested pbMECs was confirmed by immunocytochemistry using the Monoclonal Anti-Cytokeratin, pan-FITC antibody (1:200 in PBS, Sigma-Aldrich, USA, F3418). Approximately 1 × 10^4^ cells were seeded per chamber of Thermo Scientific™ Nunc™ Lab-Tek™ II CC2™ Chamber Slide System and cultured (5% CO_2_, 37 °C) in the culture media described above until confluency. Cells were first washed three times with PBS and after washing, −20 °C methanol was used for 2 min to fix the cells. Unspecific blocking of the antibody was done with 2% BSA-PBS solution with 30 min incubation at RT. After this step, cells were washed three times with PBS. Antibody solution was added to half of the chambers while PBS was added to the rest of the chambers, whereafter the chambers were incubated at RT and dark for 30 min. After incubation, cells were washed three times with PBS. Imaging was performed with an EVOS Cell Imaging System (Thermo Fisher Scientific, USA) using Evos light cube GFP and light cube DAP.

### Pathogen challenge

The challenge experiment was conducted with a Finnish strain of *Escherichia coli* FT238 isolated from bovine clinical mastitis [16]. Bacteria were cultured in Luria Bertani medium and heat-inactivated with the method described by Danowski et al. [17] and Griesbeck-Zilch et al. [18].

Then, pbMECs were thawed and seeded on 100 × 20 mm culture dish (Greiner Bio-One, Austria, collagen I coated). They were grown until ~80% confluency in an antimicrobial-free medium consisting of DMEM F12-Ham (Biowest, France) supplemented with FBS (Gibco, Thermo Fisher Scientific, USA) and ITS (Pan-Biotech, Germany). Cells were detached and ~1 × 10^5^ cells/well were seeded on in total of 30 wells per animal on 96-well plates (Greiner Bio-One, Austria, collagen I coated) and grown overnight. Next day, bacteria solution of *E. coli* [MOI (multiplicity of infection) = 10] was added into 12 wells per animal. In addition, 18 wells were kept bacteria free in a separate plate to serve as control. After 3 h and 24 h exposure to *E. coli*, six wells were harvested by adding 200 µL of 1:1 solution of antimicrobial free culture media and RNAprotect Cell Reagent (Qiagen, Germany). In addition, at timepoints 0 h, 3 h, and 24 h, six control wells were harvested as described above. Harvested cells were frozen at −80 °C.

### RNA extraction, library preparation and RNA sequencing

RNA was extracted from the three cell samples per time point per animal (three samples were kept as backup samples) using Rneasy Plus Micro kit (Qiagen, Germany) following manufacturer’s protocol. RNA quantity and quality was measured with 2100 BioAnalyzer instrument (Agilent Technologies, USA) and 50 ng of total RNA per sample was used as input for ribosomal RNA depletion using RiboCop V1.3 (Lexogen, Austria). CORALL Total RNA-Seq Library Prep Kit (Lexogen, Austria) was used for library preparation of 15 samples per animal. Library preparation was done according to manufacturer’s protocol. The quality of pooled libraries from all the animals was first checked on an Illumina iSeq100 platform at the Natural Resources Institute Finland. Three biological/technical replicates per timepoint and treatment per animal were sequenced with 2 × 150 bp read length on an Illumina NovaSeq 6000 platform at the Finnish Functional Genomics Centre (Turku, Finland).

### Data analysis

Adapter sequences and low-quality reads and bases were trimmed from the raw sequencing reads using fastp [19] with default settings. The quality before and after trimming was then evaluated with FastQC as well as MultiQC [20]. The quality trimmed reads were aligned against the *Bos taurus* reference genome ARS-UCD1.2 (Ensembl 100) with STAR [21] guided by the corresponding Ensembl 100 annotation. Read count quantification on the gene level was performed via FeatureCounts [22]. The entire data processing workflow is implemented in Snakemake [23] and is freely available as Snakebite-RNAseq pipeline [24].

### Differential expression analysis

Post-processing analyses were performed using R Statistical Software (version 4.3.1) and the *tidyverse* package [25]. Exploratory data analysis (data not shown) was performed using the *vsn* package (version 3.68.0) [26] for variance stabilisation, *pheatmap* (version 1.0.12) [27] for heat maps of sample distances and clustering. For data visualisation the packages *ggplot2* (version 3.4.3) [28], *ggrepel* (version 0.9.3) [29], *patchwork* (version 1.1.2) [30] and *RColorBrewer* (version 1.1.3) [31] were used to improve plotting, while *knitr* (version 1.44) [32] created reports.

*DESeq2* package (version 1.40.2) [33] was used to test for differential expression of genes. Minimal pre-filtering was applied to ensure that at least 3 samples had a read count of at least 10 reads. For each time point challenged samples (3 animals) were compared against control samples. The detection and treatment of count outliers was performed by *DESeq2* as previously described [33]. The automatic independent filtering provides multiple testing adjustment using the Benjamini–Hochberg method; the False Discovery Rate (FDR) cut-off alpha was set to 0.05 to identify differentially expressed genes. Volcano plots were created with EnhancedVolcano (version 1.18.0) [34].

### Annotation and enrichment analysis

Annotation of expressed genes was performed with *biomaRt* (version 2.56.1) [35] using the btaurus_gene_ensembl dataset of the bovine genome version ARS-UCD1.2. *ClusterProfiler* (version 4.8.3) [36] was used for over-representation analysis of the expressed gene sets. Enrichment of GO (gene ontology) categories for the ontology biological properties (enrichGO function) were calculated using the *org.Bt.eg.db* package (version 3.17.0) as organism database. *P*-values were adjusted for FDR using the BH (Benjamini-Hochberg) method (q-value cut-off of 0.01) was applied to report enrichment tests as significant). Dotplots showing the most significant enriched GO terms were generated using the *enrichplot* package (version 1.20.3) [37]. The linkage of genes and the GO terms as biological concept was visualised as network using the *cnetplot* function of *enrichplot*.

Over-representation analysis of Kyoto Encyclopedia of Genes and Genomes (KEGG) [38] pathways was done using the *enrichKEGG* function of *clusterProfiler* for differentially expressed genes (adjusted *p*-value < 0.05) and visualised as barplots. Based on the significant terms identified selected candidates were rendered as pathway maps using the *pathview* package (version 1.40.0) [39]. The graphs were generated based on all expressed genes and gene symbols were coloured according to fold change.

## Results

**Figure 1:**
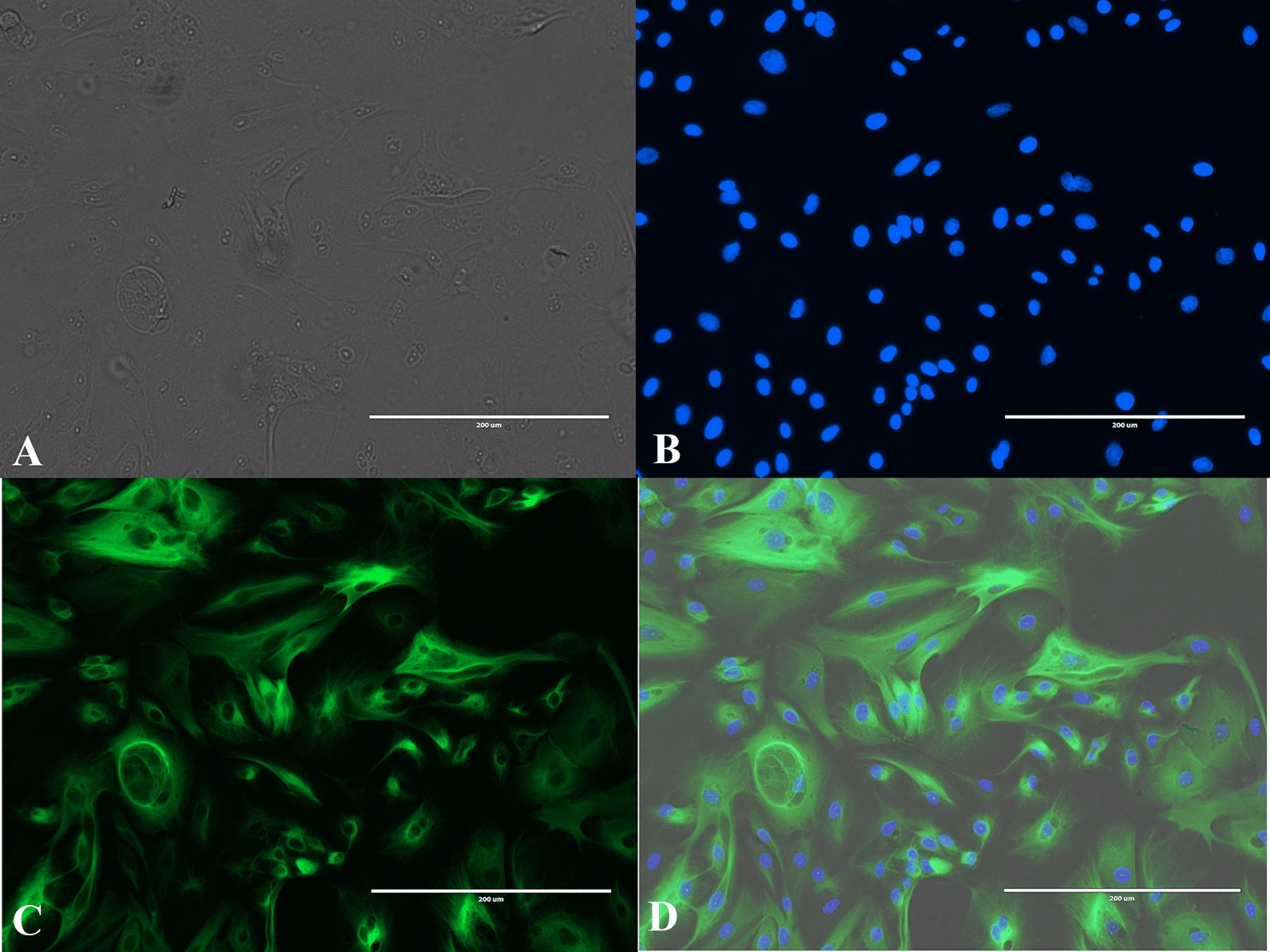
**The epithelial character of the harvested pbMECs confirmed by immunocytochemistry.** The **A** Cultured pbMECs under a light microscope, **B** Same pbMEC stained with DAPI staining under fluorescence microscopy, **C** cytokeratin staining of the pbMEC using Monoclonal Anti-Cytokeratin, pan − FITC antibody, **D** A merged image of the light microscope, DAPI and cytokeratin staining images. The bars represent the length of 200 µm.

Bovine primary mammary epithelial cells were successfully derived from fresh milk and cell cultures were formed. The epithelial character of the cells was verified by using the monoclonal anti-cytokeratin pan-FITC antibody (Figure 1).

### RNA sequencing

We sequenced three replicates of the control samples (timepoints 0 h, 3 h and 24 h) and three replicates of the challenged samples at two timepoints (3 h and 24 h) post-challenge (accession PRJEB62185). Details on data creation, alignment and gene assignment are shown in Table 1.

**Table 1:** **The RNA-Seq results showing average number of reads (M), aligned reads (%) and mapped reads (% assigned, multimapped, no feature (= no intersection with the bovine genome annotation) per treatment group**

Group	Avg. raw/trimmed reads (M reads)	Avg. aligned reads (in %)	Avg. assigned/multimapped/NoFeature (in %)
CTRL 0 h	77.6/77.0	70.0	52.91/14.13/9.18
CTRL 3 h	81.8/81.1	66.0	46.01/18.73/10.2
CTRL 24 h	88.7/88.0	69.0	49.62/17.93/9.71
*E. coli* 3 h	101.8/101.0	71.1	52.62/14.75/10.15
*E. coli* 24 h	82.0/81.3	71.4	52.0/18.79/9.07

### Differentially expressed genes at three and 24 h without E. coli challenge

We investigated the differences in gene expression among the cells (initially from same samples) that were not challenged with the *E. coli*, cultured in same conditions for 0 h, 3 h and 24 h (n for each time point was 3). From 0 h vs 3 h, there were 248 differentially expressed genes (DEGs) of which 172 were downregulated and 75 upregulated (Additional file 1). From 0 h vs 24 h, 238 DEGs were found, 127 being upregulated and 111 downregulated (Additional file 1). Four significantly enriched [*p* < 0.05 (*P*-value) and q < 0.01 (FDR)] GO terms (“steroid biosynthetic process”, “cholesterol biosynthetic process”, “secondary alcohol biosynthetic process”, and “sterol biosynthetic process”) were only found for 0 h vs 24 h (Additional file 1). Only two KEGG pathways were enriched for 0 h vs 24 h, Steroid biosynthesis (Lipid metabolism) and Ribosome biogenesis in eukaryotes (Translation, Additional file 1).

### Differentially expressed genes at three and 24 h after E. coli challenge

**Figure 2:**
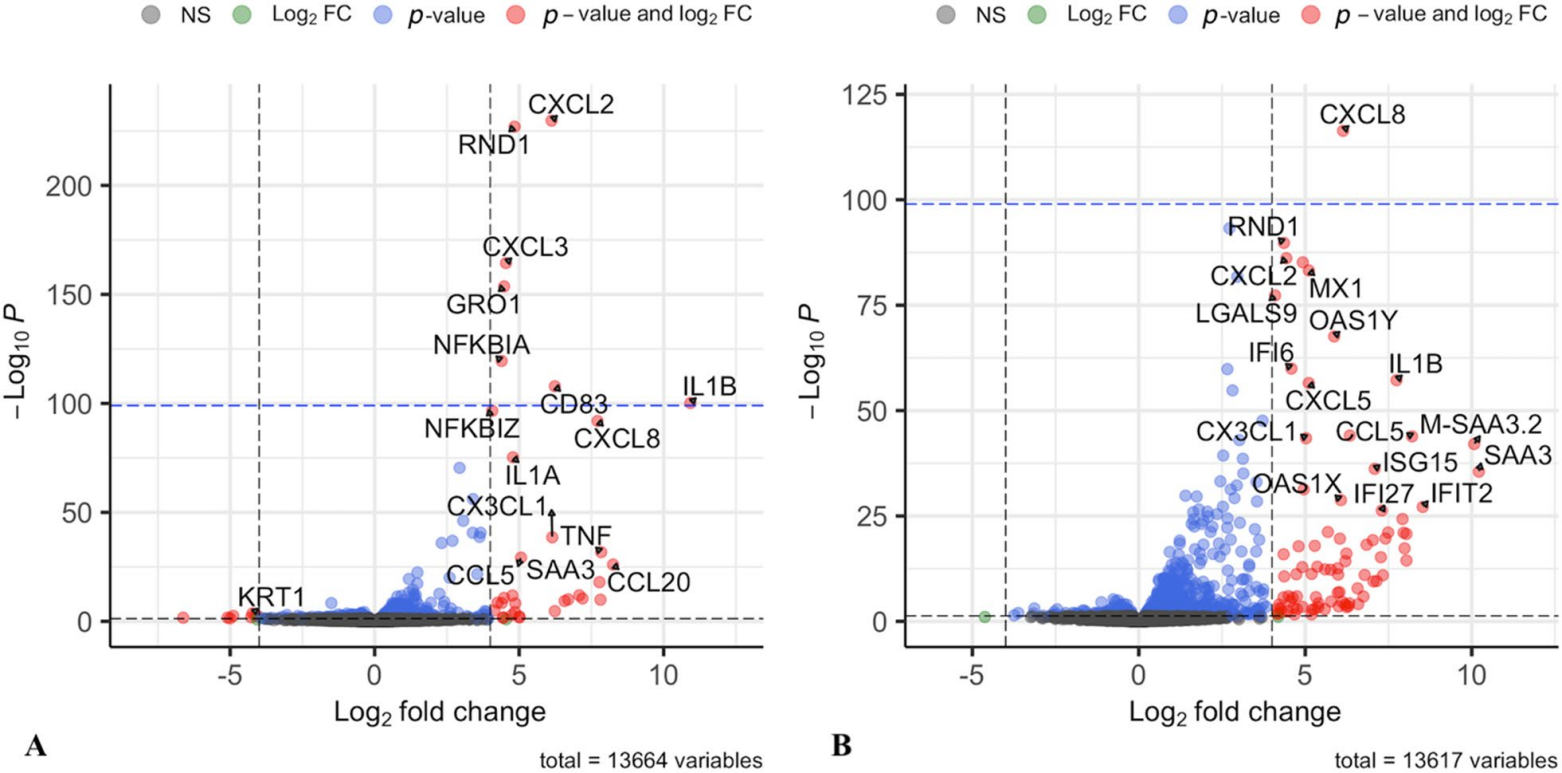
**Volcano plot showing statistical significance vs magnitude of log**_**2**_** fold change.** The genes with Log_2_ fold change > ± 5 are indicated in red, **A** 3 h post *E. coli* challenge and **B** 24 h post *E. coli* challenge. Dashed lines (vertical and horizontal) are at Log_2_ fold change = 4 and at − Log_10_ *P* = 100).

In total, 150 genes were differentially expressed 3 h post *E. coli* challenge compared to the control samples (adj. *p* < 0.05) (Table 2, Figure 2A, Additional file 2). A majority of the genes were upregulated (141 genes). At 24 h post-challenge, 440 genes were differentially expressed compared to the control samples at 24 h (adj. *p* < 0.05) (Table 2, Figure 2B, Additional file 2). Also, at 24 h, most of the DE genes were upregulated (*n* = 424). The top 10 differentially expressed genes (DEGs) at 3 h and 24 h are shown in Table 3. Altogether 89 genes were upregulated both at 3 h and 24 h post-challenge whereas, in comparison, no genes were downregulated at both timepoints (Additional file 3).

**Table 2:** **The numbers of differentially expressed genes (DEGs) at 3 h or 24 h post** ***E. coli*** **challenge**

	3 h post *E. coli* challenge	24 h post *E. coli* challenge
	n (P_adj_ < 0.05)	n (P_adj_ < 0.05)
All	150	440
Upregulated	141	424
Downregulated	9	16

*n* = number of genes.

**Table 3:** **The top 10 differentially expressed genes at 3 h and at 24 h post *E. coli*** **challenge**

Gene	ID	Log_2_ Fold Change	P_adj_	Gene	ID	Log_2_ Fold Change	P_adj_
3 h post* E. coli* challenge							
*CXCL2*	ENSBTAG00000027513	6.12	2.94 × 10^−−226^	*IL1B*	ENSBTAG00000001321	10.92	1.38 × 10^–97^
*RND1*	ENSBTAG00000018773	4.84	8.73 × 10^−−224^	*CCL20*	ENSBTAG00000021326	8.25	5.95 × 10^–24^
*CXCL3*	ENSBTAG00000037778	4.55	4.55 × 10^–161^	*TNF*	ENSBTAG00000025471	7.84	1.29 × 10^–29^
*GRO1*	ENSBTAG00000037558	4.48	8.6 × 10^–151^	*SLC6A12*	ENSBTAG00000038415	7.82	4.06 × 10^–08^
*NFKBIA*	ENSBTAG00000016683	4.39	1.05 × 10^–116^	*SAA3*	ENSBTAG00000049589	7.78	5.72 × 10^–16^
*CD83*	ENSBTAG00000050334	6.24	3.43 × 10^–105^	*CXCL8*	ENSBTAG00000019716	7.71	2.01 × 10^–89^
*IL1B*	ENSBTAG00000001321	10.92	1.38 × 10^–97^	*CCL4*	ENSBTAG00000025257	7.19	1.12 × 10^–08^
*NFKBIZ*	ENSBTAG00000010987	4.07	4.91 × 10^–94^	*IFNL3*	ENSBTAG00000050296	7.08	4.94 × 10^–10^
*CXCL8*	ENSBTAG00000019716	7.71	2.01 × 10^–89^	*IFIT2*	ENSBTAG00000034918	6.70	2.86 × 10^–08^
*IL1A*	ENSBTAG00000010349	4.79	7.99 × 10^–73^	*M-SAA3.2*	ENSBTAG00000054278	6.57	1.46 × 10^–07^
24 h post* E. coli* challenge							
*CXCL8*	ENSBTAG00000019716	6.14	6.82 × 10^–113^	*SAA3*	ENSBTAG00000049589	10.22	1.99 × 10^–33^
*NFKBIA*	ENSBTAG00000016683	2.72	4.54 × 10^–90^	*M-SAA3.2*	ENSBTAG00000054278	10.07	6.58 × 10^–40^
*RND1*	ENSBTAG00000018773	4.36	8.85 × 10^–87^	*IFIT2*	ENSBTAG00000034918	8.53	2.92 × 10^–25^
*CXCL2*	ENSBTAG00000027513	4.43	2.85 × 10^–83^	*CCL5*	ENSBTAG00000053649	8.21	1.19 × 10^–41^
na	ENSBTAG00000045588	4.93	2.11 × 10^–82^	*MX2*	ENSBTAG00000008471	8.04	4.53 × 10^–19^
*MX1*	ENSBTAG00000030913	5.11	1.53 × 10^–80^	*SAA2*	ENSBTAG00000022394	8.04	5.90 × 10^–13^
*RIGI*	ENSBTAG00000003366	2.96	4.06 × 10^–79^	*GBP2*	ENSBTAG00000038938	7.98	9.72 × 10^–16^
*LGALS9*	ENSBTAG00000006846	4.09	9.75 × 10^–75^	*TNFRSF9*	ENSBTAG00000003313	7.96	2.37 × 10^–19^
*OAS1Y*	ENSBTAG00000039861	5.87	4.45 × 10^–65^	*IFIT3*	ENSBTAG00000009768	7.92	1.78 × 10–^22^
*IFI6*	ENSBTAG00000007554	4.58	1.68 × 10^–57^	*IL1B*	ENSBTAG00000001321	7.74	7.78 × 10^–55^

The top 10 genes are listed based on both adjusted *P* value (P_adj_) and Log_2_ Fold Change.

### Enriched GO terms and KEGG pathways at 3 h and 24 h post E. coli challenge

When the cut-off for enriched GO terms [*p* < 0.05 (*P*-value) and q < 0.01 (FDR)] were applied, 244 and 176 GO terms for the timepoints 3 h and 24 h post-challenge, respectively, remained. Of those, 132 were the same for both timepoints and 112 (3 h) and 44 (24 h) unique (Additional files 3, 4). The most significant GO biological process at 3 h and 24 h post-challenge was the “immune system process” (GO:0002376). Similarly, the two next significantly enriched terms were “immune response” and “defense response” for both timepoints. Specifically, among the 20 most significant GO biological processes in addition to cytokine mediated processes at 3 h there are several T-cell activation related processes, whereas at 24 h interspecies interaction and response to other organisms and external stimulus are frequent.

**Figure 3:**
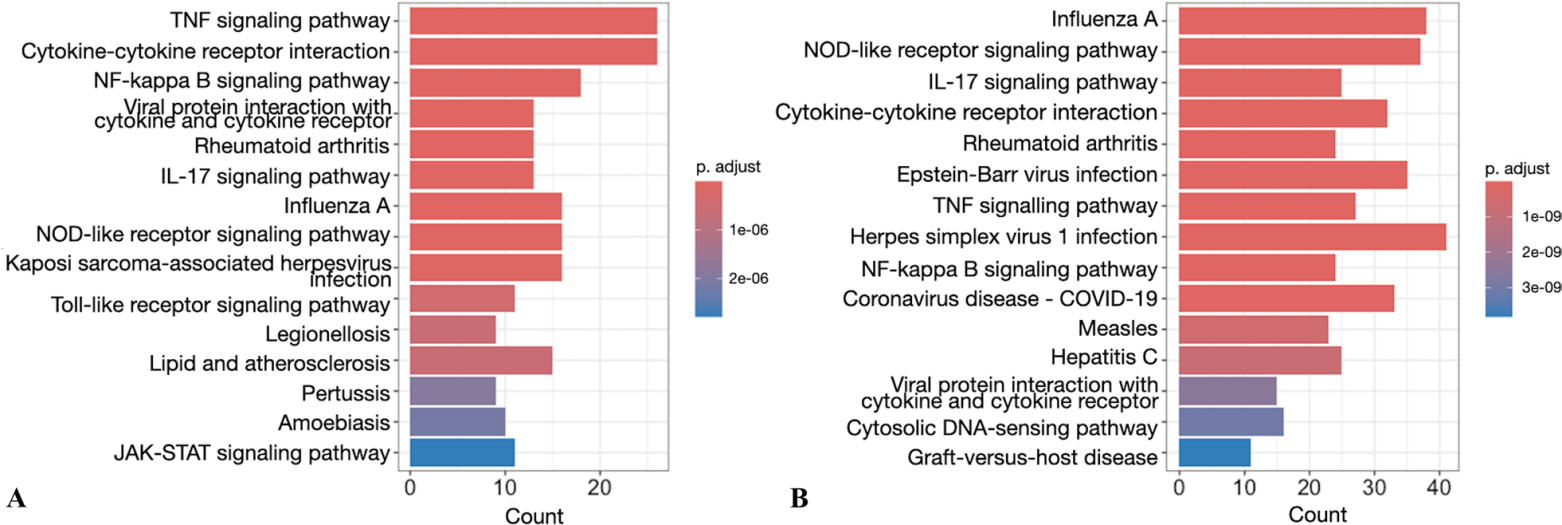
**Top 15 enriched KEGG pathways.** **A** at 3 h and **B** 24 h post-challenge. The colour coding indicates adjusted *p*-value and the count is the number of DE genes annotated in the specific pathway.

KEGG pathway analysis (using default settings) resulted in 60 enriched pathways at 3 h and 61 enriched pathways at 24 h post-challenge (Additional file 5). Of these enriched pathways, 45 were shared between the timepoints, 15 were unique for 3 h post-challenge, and 16 unique for 24 h post-challenge. The most significant 15 pathways for both timepoints are shown in Figure 3. From the highly significant enriched pathways at 24 h post-challenge, one is unique for this timepoint (“bta04612 Antigen processing and presentation”, Additional file 5). Altogether 8 pathways are shared among the top 15 pathways enriched after 3 h and 24 h.

The TNF signaling pathway was highly significantly enriched in cells both at 3 h and 24 h post-challenge (Figure 3) and abundant among top 10 DE genes (Table 3). The strong inflammatory response presented by induction of pro-inflammatory cytokines TNF-alpha and IL1B at 3 h post-challenge was balanced by upregulation of immune-dampening factors such as the two NF-kappaB inhibitors NFKBIA and NFKBIZ. Some changes in gene expression in the pathway were seen during the experiment (Additional file 6). At 3 h the upregulation is clear in genes related to leucocyte recruitment (*CCL5*, *CCL20*, *CXCL1* = *GRO1*, *CXCL2*, *CXCL3*, *CXCL5*, and *CX3CL1*), leukocyte activation (CSF2), inflammatory cytokines (*IL1B*, *IL6*, *TNF*) and negative regulation of intracellular signaling (*NFKBIA*, *NFKBIZ*, *TNFAIP3*). At 24 h the upregulation in leukocyte recruitment, leucocyte activation and inflammatory cytokines genes remains, but negative regulation of intracellular signaling is less upregulated (not *TNFAIP3*). In addition, a gene for remodeling of extracellular matrix (MMP3) is upregulated. Furthermore, IRF1 and IFNbeta are upregulated at 3 h, but not differentially expressed at 24 h. The upregulation of IFNalpha/beta at 3 h and no differential expression at 24 h is also seen in the “NOD-like receptor signaling pathway” that was the second most significantly enriched pathway at 24 h post-challenge and also among the top 20 enriched pathways at 3 h. At 24 h the oligoadenylate synthase family member *OAS1Y* was one of the top 10 upregulated genes. The *OAS1Y* gene is reported to be one of the core genes in the “NOD-like receptor signaling pathway” [40]. The antigen processing and presentation pathway specifically enriched at 24 h shows the strong upregulation of MHCI and MHCII loci at 24 h, in contrast to 3 h (Additional file 6).

## Discussion

Bovine mastitis is still a major economical and welfare issue in the dairy industry. One way to combat this problem is to breed cows to be more resistant to mastitis. Here we propose a non-invasive, in vitro method to investigate responses of udder epithelial cells against mastitis pathogens. Gene expression of the primary mammary epithelial cells without pathogen challenge was not affected during the experimental period similarly as challenged pbMECs gene expression. The few DE genes in the control cells are enriched in two KEGG pathways: “Steroid biosynthesis (Lipid metabolism)” and “Ribosome biogenesis in eukaryotes”. Our post-challenge results are in line with the known effects of *E. coli* udder infection: early strong inflammatory response mediated via pathogen receptors like the TLR and NOD families that activate the TLR signaling cascade and ultimately NF-kappaB, which are known master regulators of immune functions and genes. At 24 h post-challenge, the innate immune system toll like receptor cascades and cytokine signaling are still enhanced and the adaptive immune system is getting highlighted by antigen presentation by MHC (major histocompatibility complex) I and II class genes. By pathogen challenge, we were thus able to repeat known early immune system responses to *E. coli* infection and also identify novel potential candidate genes for further studies. We suggest that variation in the response may provide a path for the identification of important genes and pathways behind mastitis resistance, as the epithelial cells of the inner surface of the mammary gland play a key role in recognizing mastitis-causing pathogens [41].

Some of the genes reported here for the first time to be differentially expressed in association with *E. coli* challenge have been reported to be involved in mastitis in other species but not in bovine. For instance, the *STEAP4* gene (STEAP4 metalloreductase, ENSBTAG00000002340) that plays a role in systemic metabolic homeostasis and integrated inflammatory and metabolic responses, has been reported to have increased expression after *S. aureus* infection in goats [42]. *IL19* (interleukin-19, ENSBTAG00000006692, Additional file 2) expression is upregulated in humans with mastitis and the gene has potentially been responsive to the treatment with the probiotic *Lactobacillus salivarium* [43]. Similarly, Li et al. [44] found that multifunctional probiotic *Lactobacillus plantarum* downregulated the mRNA expression levels of *LR2*, *TLR4*, *MyD88*, *IL1B*, *IL6*, *IL8*, *TNFα*, *COX2*, *iNOS*, *CXCL2* and *CXCL10* genes in *E. coli* induced inflammatory responses of mammary epithelial cells. From those, *IL1B*, *TNFα*, and *CXCL2* are also among the top 10 upregulated genes in this study (Table 3). The European Union One Health Action plan against antimicrobial resistance calls for alternative treatments in livestock farming systems to support good animal health and welfare. Our results indicate these as potential targets to reduce the use of antimicrobials in the livestock industry.

Attempts to improve mastitis resistance through conventional breeding have not been very successful due to low heritability of clinical mastitis, difficulties in recording and unfavourable genetic correlations with milk yield [45]. Several studies have attempted to identify potential gene targets for breeding by whole genome association approach or studying gene expression differences in connection of mastitis in vivo or in vitro. However, the results have varied a lot between studies, and so far, there is no clear understanding of the most favourable genomic targets for breeding. In the recent review by Brajnik and Ogorevc [11], a data integration approach was used to identify the most promising candidate loci for mastitis resistance. In all, they identified 2300 genes (2287 having a ENSEMBL ID) that were differentially expressed during mastitis in nine different transcriptome-based studies. When comparing our results to the gene list of Brajnik and Ogorevc [11], we found that at 3 h post-challenge, 80 of all DE genes in our study were not on the list. At 24 h post-challenge, 229 of all DE genes (37 overlapping with those at 3 h) were not included in the DE genes listed by [11]. So, in all, our study indicates a total of 272 genes as potential novel targets for mastitis studies.

Most of the downregulated genes, (8/9 at 3 h and 13/16 at 24 h post-challenge) have not been reported previously as differentially expressed after pathogen challenge (Additional file 7). This may indicate that using RNA-Seq downregulated genes are detected more efficiently, or that downregulated genes are not typically reported, or that our study setup (control samples always taken at the same time post-challenge as the challenged samples) allows more efficient detection of also downregulated genes. Based on their literature review, Brajnik and Ogorevc [11] listed 22 genes being the most promising candidate genes for mastitis resistance. One of those [*CXCL8* (ENSBTAG00000019716)] is upregulated in our study at both 3 h and 24 h. *CXCL8* as a candidate gene is highly supported by other studies as well (27 references listed in [11]). In addition, *LTF* (ENSBTAG00000001292) is upregulated at 24 h.

In conclusion, pbMEC extracted from milk can serve as a tool to measure innate immune responses against various pathogens. Our results confirm the specific activation of genes and pathways after *E. coli* challenge thereby enabling detailed studies on sequence variations causing differences between resistance phenotypes of cattle. Distinguishing between causative and neutral variants is essential for biologically informed genomic prediction and selection of dairy cattle, expected to be more effective than the current breeding technologies [46]. Our results pave the way for studying individual differences and also their connection with genetic variation in important regulatory elements.

### Supplementary Information

**Additional file 1. Statistically significantly (P**_**adj**_** < 0.05) differentially expressed genes.** GO terms and KEGG pathways at 0 h vs 3 h and at 0 h vs 24 h control cells.

**Additional file 2. Statistically significantly (P**_**adj**_** < 0.05) differentially expressed genes at three hours and 24 h.**

**Additional file 3. Venn diagrams to illustrate overlapping DEGs, GO terms and KEGG terms between the timepoints.** A) Venn diagram of the DEGs at 3 h and 24 h timepoints. B) Venn diagram of the GO terms at 3 h and 24 h timepoints c) Venn diagram of the KEGG terms at 3 h and 24 h timepoints.

**Additional file 4. Enriched GO terms for 3 h and 24 h after *E. coli* pathogen challenge.**

**Additional file 5. Enriched KEGG pathways for 3 h and 24 h after *E. coli* pathogen challenge.**

**Additional file 6. Changes in gene expression along the time in the TNF signaling pathway, Influenza A and Antigen processing and presentation pathways.** KEGG pathway maps for the A) TNF signaling pathway, (bta04667), left: 3 h post-challenge and right: 24 h post-challenge B) Influenza A (bta05164), left: 3 h post-challenge and right: 24 h post-challenge and C) Antigen processing and presentation pathway (bta04612), left: 3 h post-challenge and right: 24 h post-challenge. The significantly differentially expressed genes are marked in red (upregulated) or green (downregulated) and shaded according to the fold change.

**Additional file 7. Mastitis associated DE genes not listed in Brajnik and Ogorevc, 2023.**

## Data Availability

The RNA-sequence data is available under ENA accession PRJEB62185.
