# Supplementary material for: Genes and pathways revealed by whole transcriptome analysis of milk derived bovine mammary epithelial cells after Escherichia coli challenge
Source: Vet Res. 2024 Feb 1;55:13. doi: 10.1186/s13567-024-01269-y (PMC10835992; doi:10.1186/s13567-024-01269-y)

**Additional file 3.** **Venn diagrams to illustrate overlapping DEGs, GO terms and KEGG terms between the timepoints** A) Venn diagram of the DEGs at 3 h and 24 h timepoints. B) Venn diagram of the GO terms at 3 h and 24 h timepoints c) Venn diagram of the KEGG terms at 3 h and 24 h timepoints.


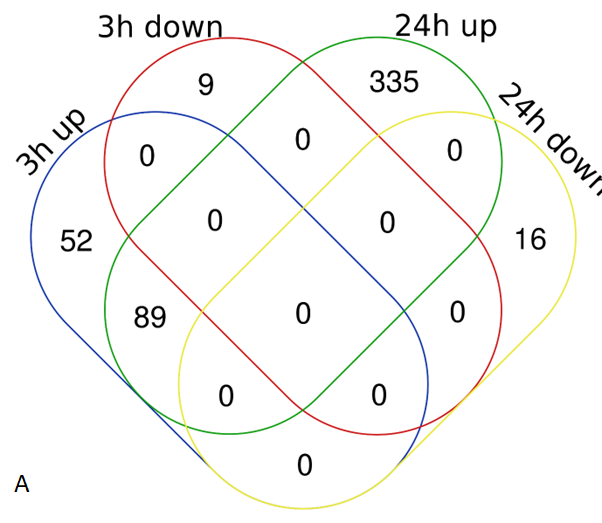


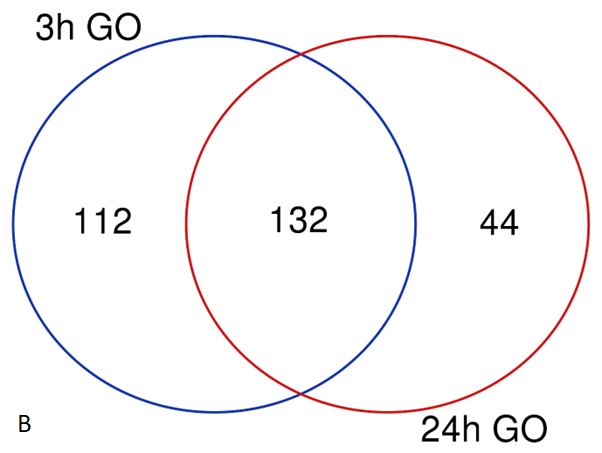


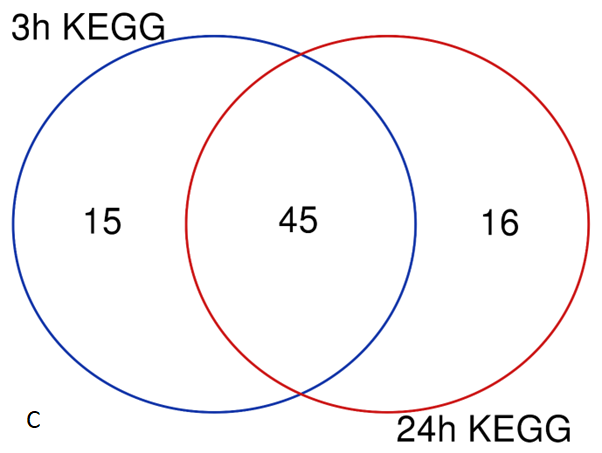

Supplement: Supplementary file 3 — Additional file 3. Venn diagrams to illustrate overlapping DEGs, GO terms and KEGG terms between the timepoints. A) Venn diagram of the DEGs at 3 h and 24 h timepoints. B) Venn diagram of the GO terms at 3 h and 24 h timepoints c) Venn diagram of the KEGG terms at 3 h and 24 h timepoints. [file 13567_2024_1269_MOESM3_ESM.docx]
