# Supplementary material for: Genes and pathways revealed by whole transcriptome analysis of milk derived bovine mammary epithelial cells after Escherichia coli challenge
Source: Vet Res. 2024 Feb 1;55:13. doi: 10.1186/s13567-024-01269-y (PMC10835992; doi:10.1186/s13567-024-01269-y)
Supplement: Supplementary file 6 — Additional file 6. Changes in gene expression along the time in the TNF signaling pathway, Influenza A and Antigen processing and presentation pathways. KEGG pathway maps for the A) TNF signaling pathway, (bta04667), left: 3 h post-challenge and right: 24 h post-challenge B) Influenza A (bta05164), left: 3 h post-challenge and right: 24 h post-challenge and C) Antigen processing and presentation pathway (bta04612), left: 3 h post-challenge and right: 24 h post-challenge. The significantly differentially expressed genes are marked in red (upregulated) or green (downregulated) and shaded according to the fold change. [file 13567_2024_1269_MOESM6_ESM.docx]

**Additional file 6. Changes in gene expression along the time in the TNF signaling pathway, Influenza A and Antigen processing and presentation pathways.** KEGG pathway maps for the A) TNF signaling pathway, (bta04667), left: 3 h post challenge and right: 24 h post-challenge B) Influenza A (bta05164), left: 3 h post challenge and right: 24 h post-challenge and C) Antigen processing and presentation pathway (bta04612), left: 3 h post-challenge and right: 24 h post-challenge. The significantly differentially expressed genes are marked in red (upregulated) or green (downregulated) and shaded according to the fold change.


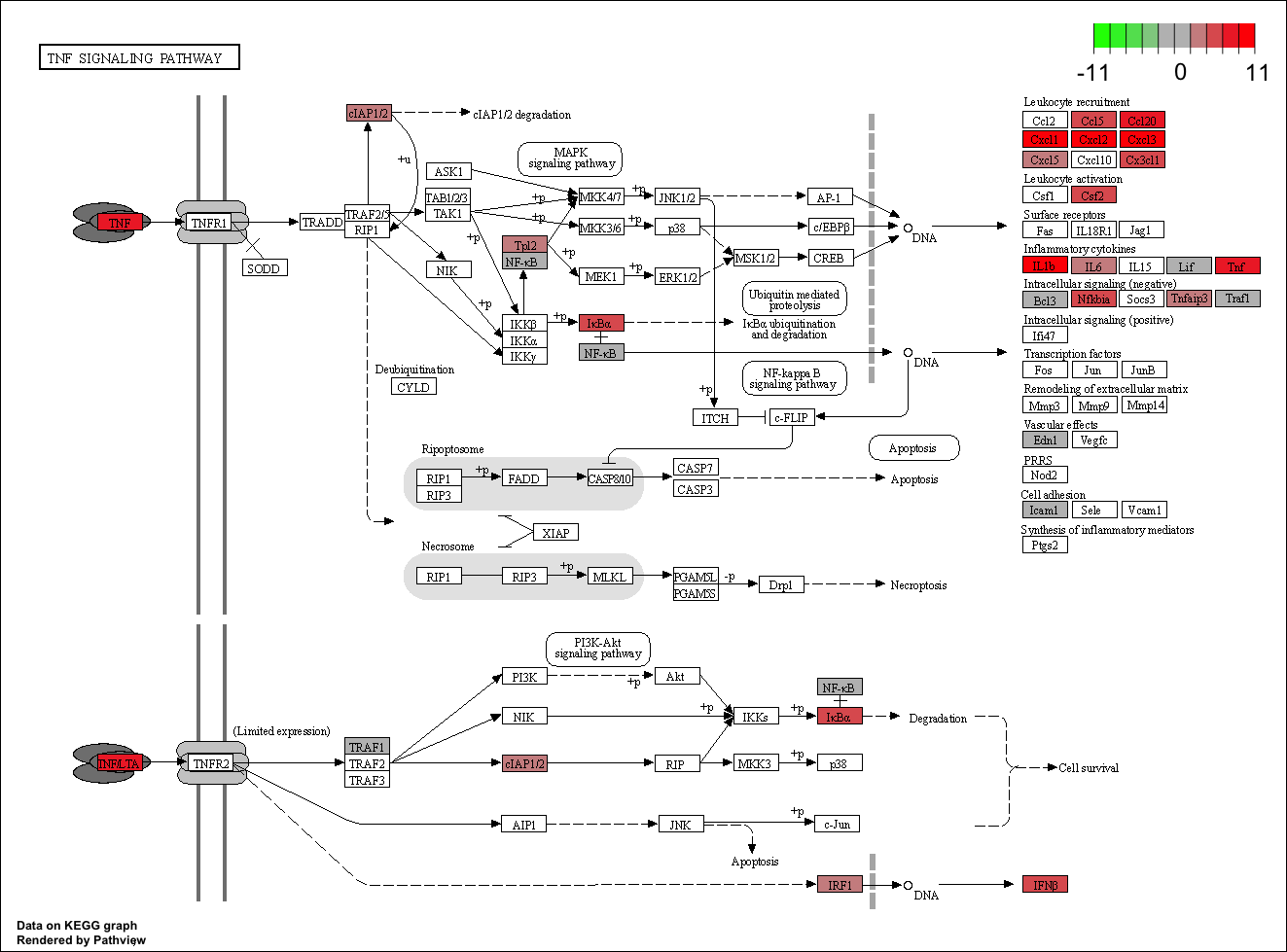

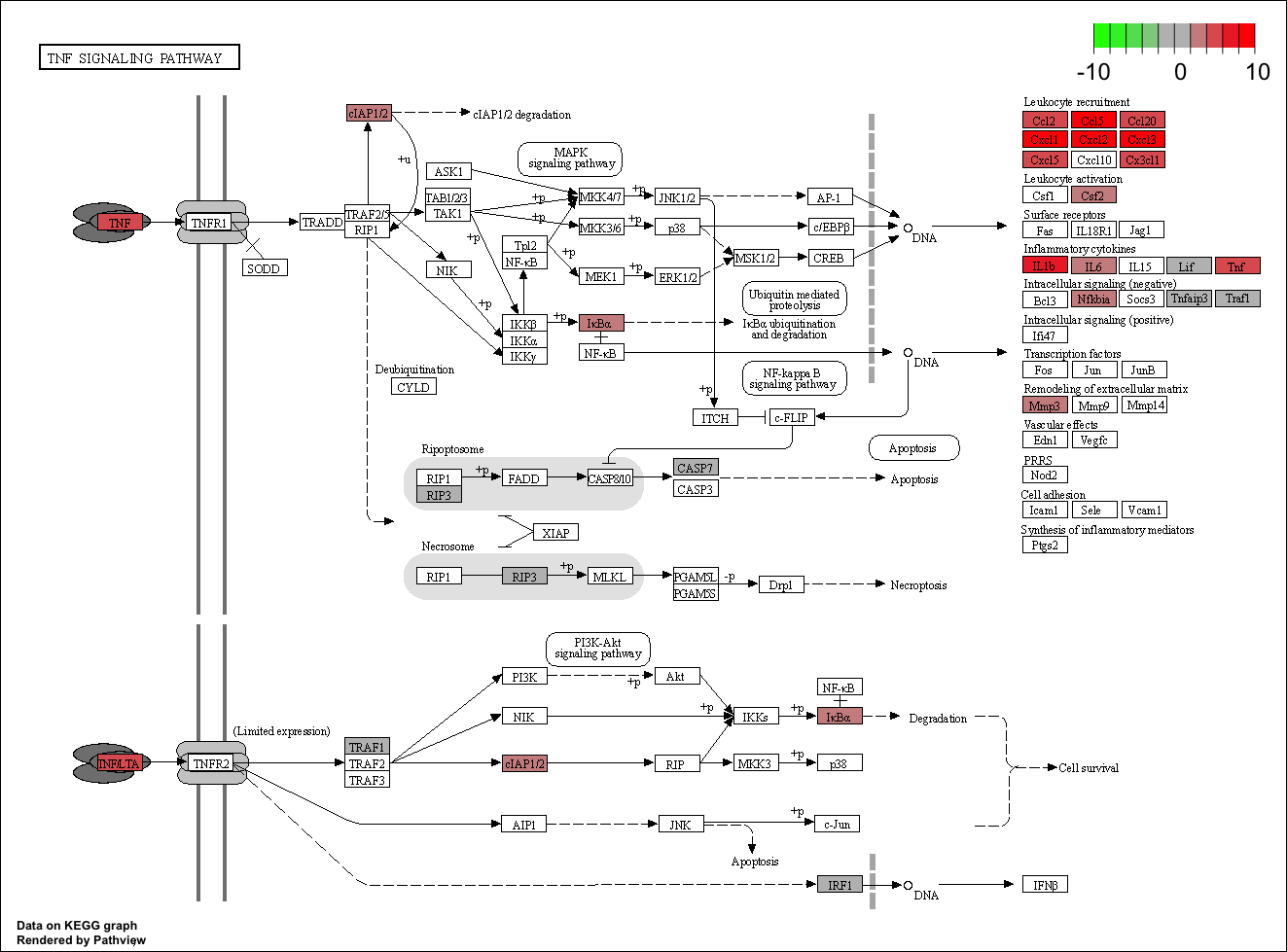


A


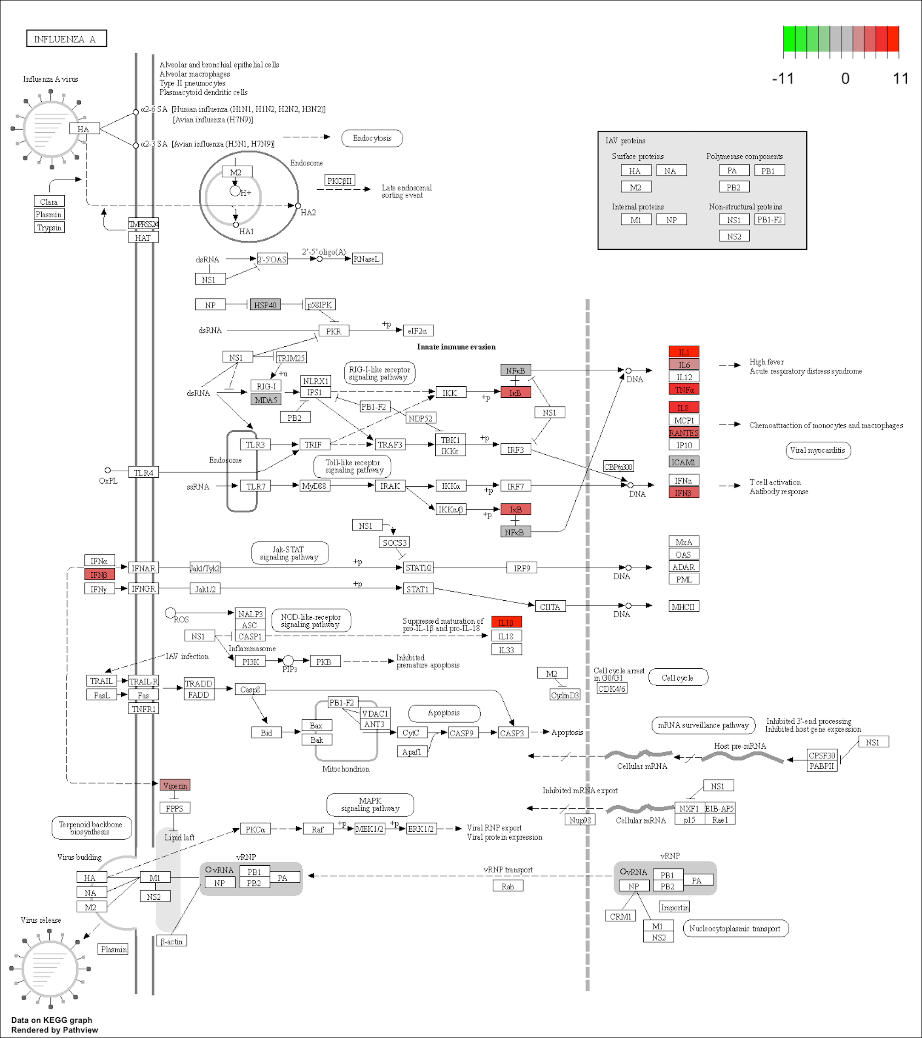

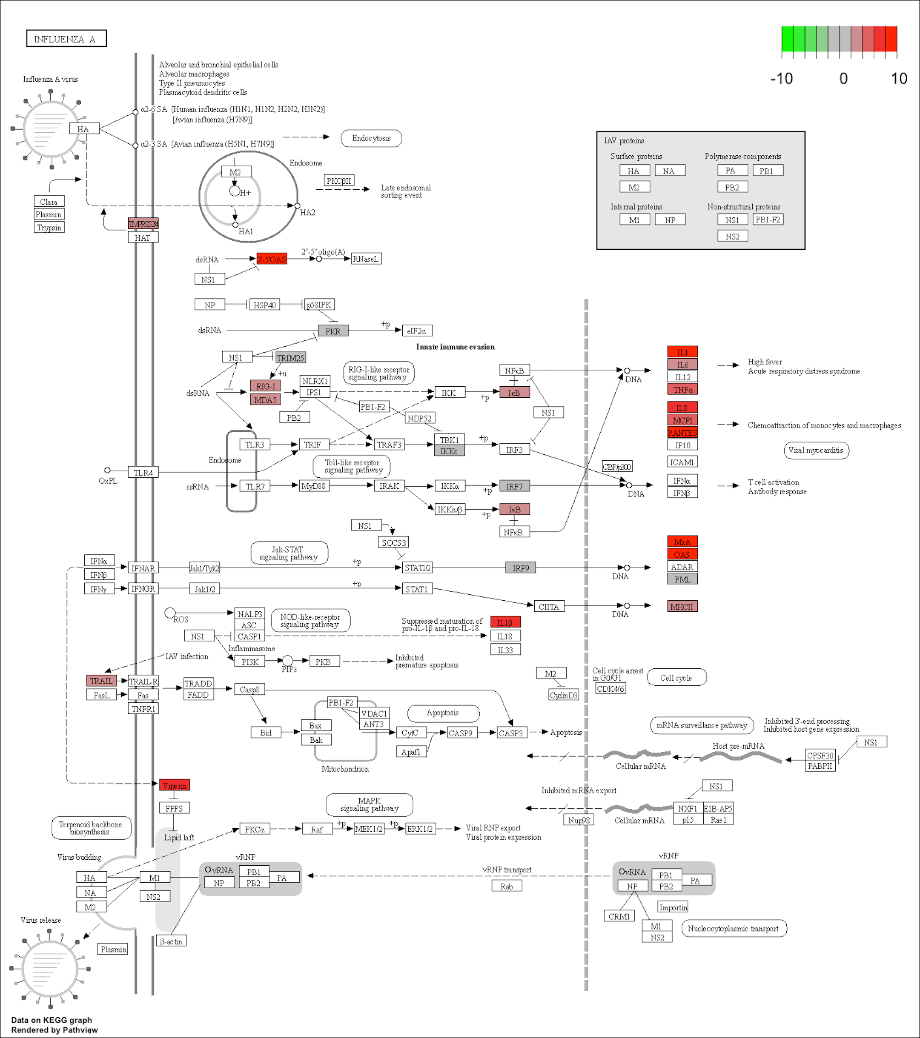


B


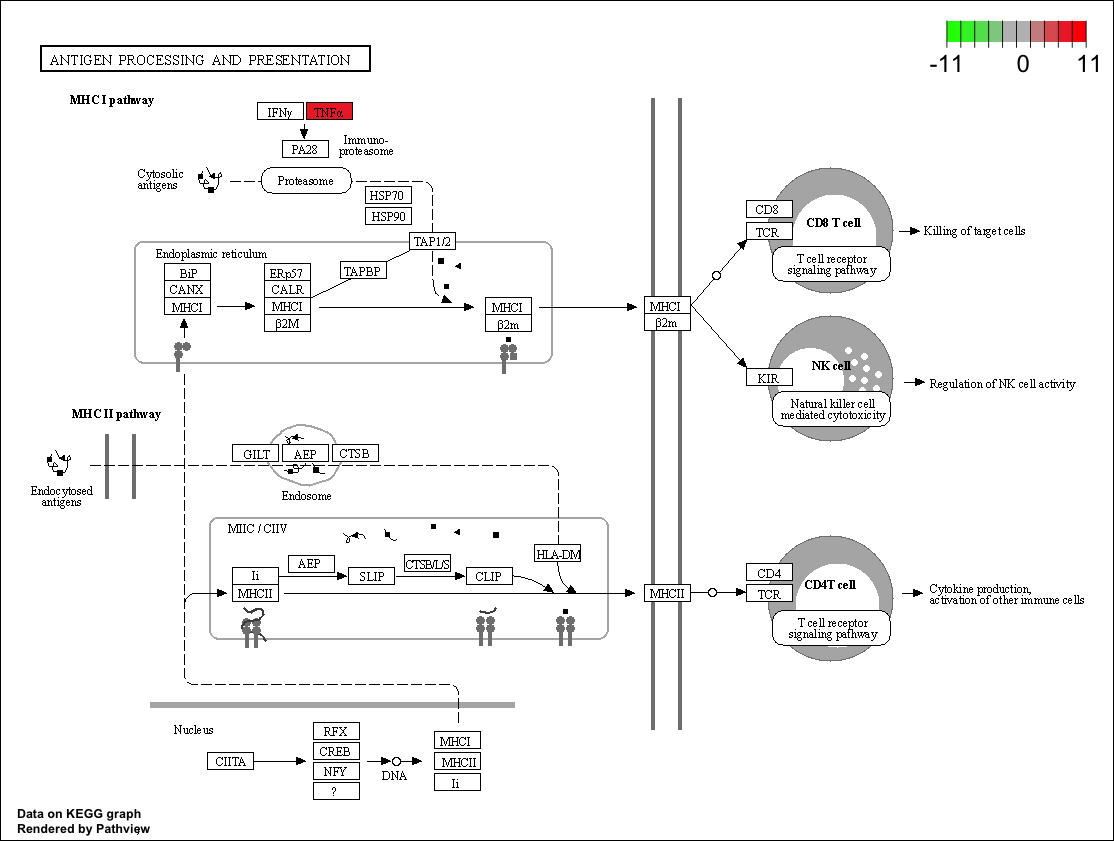

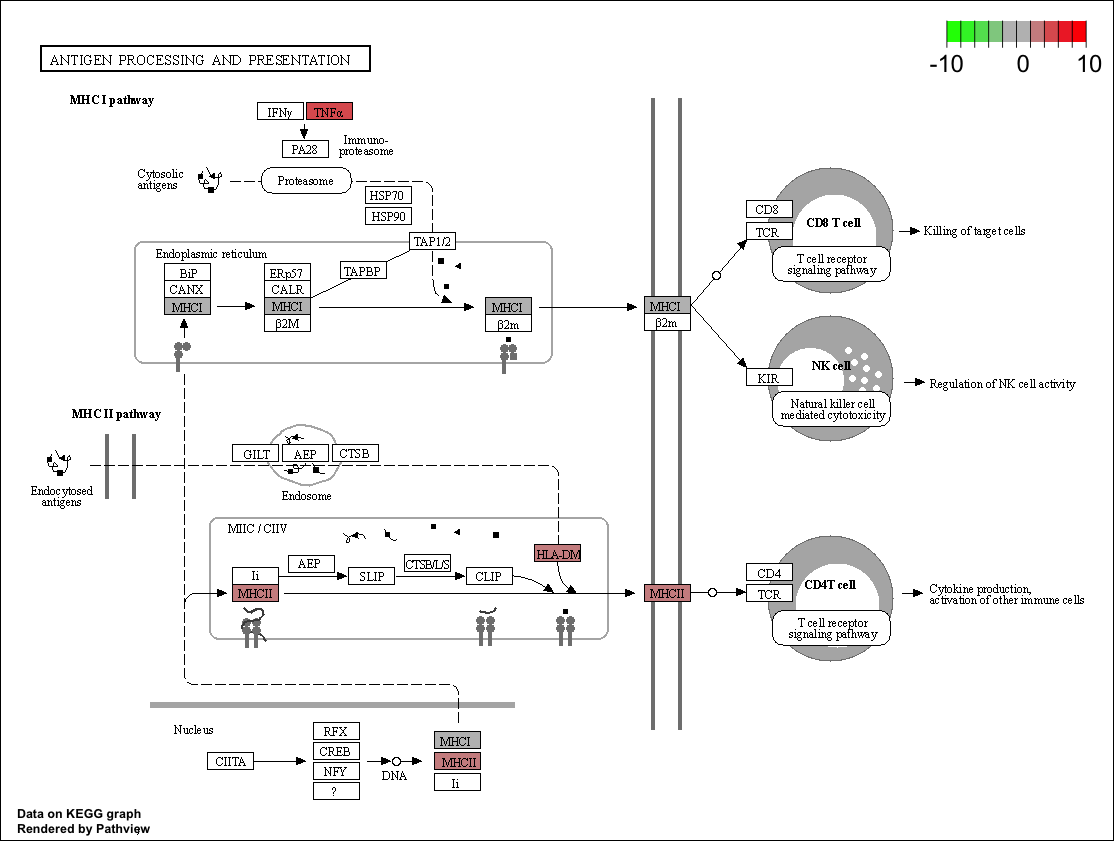


C
